# The High-Throughput Analyses Era: Are We Ready for the Data Struggle?

**DOI:** 10.3390/ht7010008

**Published:** 2018-03-02

**Authors:** Valeria D’Argenio

**Affiliations:** 1 CEINGE-Biotecnologie Avanzate, via G. Salvatore 486, 80145 Naples, Italy; dargenio@ceinge.unina.it; Tel.: +39-081-3737909; 2 Department of Molecular Medicine and Medical Biotechnologies, University of Naples Federico II, via Pansini 5, 80131 Naples, Italy

**Keywords:** high-throughput analysis, next-generation sequencing, big data, -omic sciences, personalized medicine

## Abstract

Recent and rapid technological advances in molecular sciences have dramatically increased the ability to carry out high-throughput studies characterized by big data production. This, in turn, led to the consequent negative effect of highlighting the presence of a gap between data yield and their analysis. Indeed, big data management is becoming an increasingly important aspect of many fields of molecular research including the study of human diseases. Now, the challenge is to identify, within the huge amount of data obtained, that which is of clinical relevance. In this context, issues related to data interpretation, sharing and storage need to be assessed and standardized. Once this is achieved, the integration of data from different -omic approaches will improve the diagnosis, monitoring and therapy of diseases by allowing the identification of novel, potentially actionably biomarkers in view of personalized medicine.

## 1. Introduction

Rapid technological advances have made high-throughput technologies available for the study of biological systems in their integrity, laying the foundation for the development of the so-called “-omics” era [1,2]. Indeed, the completion of the first human genome sequence draft [3,4] and the availability of high-scale technological tools have made it possible to study genomics, transcriptomics, epigenomics, and other -omic sciences at a previously unthinkable level [2,5,6,7]. The integration of these disciplines is increasing our understanding of the molecular bases of human diseases (both acquired and inherited), with the final aim to improve their diagnosis, monitoring, and treatment, in view of an even more personalized medicine [2]. The currently available technologies can generate gigabytes of data per day with a good level of accuracy and reliability [2,5,6,7]. This feature has pushed molecular research beyond the limitations imposed by the more traditional analytical approaches. However, it was soon clear that the same feature badly conceals an important side effect: high-throughput technologies generate a high quantity of data, whose management, analysis and storage require specific infrastructures and bioinformatic pipelines [8,9]. In particular, the correct interpretation of these data to extrapolate, from the huge amount of information, only that which is relevant from a clinical point of view, represents nowadays a great challenge [10]. Also, ethical issues related to incidental findings, data property, and privacy aspects are animating scientific debates and need to be carefully regulated to avoid risks related to the data struggle.

## 2. High-Throughput Analyses

The last 15 years have featured the development and the fast diffusion of next-generation sequencing (NGS) technologies [2,5,6,7]. These techniques have impacted every field of molecular research, escalating previously used sequencing technologies [11], and opening the way to the -omic sciences foundation [1,2]. Indeed, NGS methods allow the sequencing of entire genomes [12,13,14,15], of exomes [16,17,18], of panels of genes related to a disease of interest [19,20,21], or of a single gene [22,23,24,25,26], but can also be used to explore the entire transcriptome [27,28,29], small RNAs [30,31,32], the epigenome [33,34], and the microbiome [35,36,37,38].

Notwithstanding some peculiar characteristics related to the different manufacturers, the currently available NGS sequencers are based on the amplification of one specific library (or multiple barcoded libraries), i.e., a pool of DNA fragments representing the target to be sequenced, on the surface of a flow cell, or of specific microscopic beads, to obtain clonal clusters of fragments that will be massively sequenced, as is extensively reviewed elsewhere [5,6,7]. Next-generation sequencing techniques combine the high-throughput capability and a high sequencing reads accuracy with a low cost per base: it is amazing to think that the cost for the sequencing of an entire human genome has dropped from about US $10 million to about US $1000 in just the last 10 years [39].

In this view, it is not surprising that NGS is also becoming a reference method for molecular diagnostics [10]. In particular, NGS allows not only the analysis, in more patients simultaneously, of disease-related genes in less time and at lower costs than traditional approaches, but also the sequencing of panels of genes up to the complete exome [16,17,18,19,20,21,22,23,24,25,26]. In this way, it is possible to increase the diagnostic sensitivity, to discover novel disease-related genes and also obtain data regarding other genes that were potentially acting as disease-phenotype modifiers [19,40,41,42]. Due to their high sensitivity and flexibility, NGS methods are also useful for prenatal and preimplantation diagnostics [43,44,45,46], and other applications, such as the sequencing of circulating free DNA or of single cells (i.e., fetal cells or circulating tumor cells), are being validated [45,47,48,49].

In addition to the study of sequence variations at the DNA level, NGS methods can be used to study genetic variability, and the mechanisms underlying the onset of specific diseases at epigenetic, transcriptomic and metagenomic levels [27,28,29,30,31,32,33,34,35,36,37,38]. Indeed, several factors, other than individual genetic predisposition, such as diet, environmental factors and lifestyle, can influence the epigenome, the transcriptome and the microbiome [2,50]. Thus, all these systems are dynamic and can feature specific modifications related to a specific pathological status. Understanding such modifications not only sheds light on the mechanisms underlying the disease development, but may also provide novel, potential biomarkers for an earlier and/or more accurate diagnosis, for the stratification of patients into prognostic classes, for disease monitoring, and/or for the development of specific and consequently more effective targeted therapies. Next-generation sequencing-based approaches, by providing both a high sequencing coverage and an unbiased view of complex systems without the need of an a priori knowledge of the targets of interest, have also imposed new analytical standards in these fields [2,7,10].

In the case of RNA studies for example, NGS-based approaches have overcome the use of microarrays: NGS allows the analysis of virtually all the RNA molecules, known and unknown, present in a sample, at a lower cost [2,7]. In addition, alternative splicing isoforms and long non-coding RNAs can also be highlighted [2,7,27,28,29], and specific small RNA classes can be enriched and sequenced [30,31,32]. Finally, recent applications are also showing the potential of NGS for single cells RNA sequencing [51,52]. Similarly, NGS has prompted the study of the epigenome and of the microbiome. By using the preparation protocols of specific libraries, it is possible to analyze the methylation status of DNA at a genome-wide level or by focusing on a custom set of genomic regions of interest [33,34]. Moreover, chromatin immunoprecipitation sequencing (ChIP-Seq) approaches have shown their efficacy in the study of the regulatory networks of gene expression at the genome-wide level, by allowing the identification of the targets of specific transcription factors [53]. Recently, the newest epigenomic methodologies, such as micrococcal nuclease sensitive sites sequencing (MNase-seq), DNase I hypersensitive sites sequencing (DNase-seq), formaldehyde-assisted isolation of regulatory elements sequencing (FAIRE-seq), and assay for transposase-accessible chromatin using sequencing (ATAC-seq), have been developed and have shown their reliability for the study of chromatin accessibility at the genome-wide level to identify the epigenetic changes responsible for differential gene expression, cell proliferation, functional diversification and disease development [54].

Finally, by superseding the need of microbial cultivation, NGS-based techniques also gave a significant boost to metagenomics for the study of the microbial relationships with human physiology and pathology, and for the identification of specific microbial signatures related to a disease of interest [35,36,37,38,50]. It is now established that the human microbiome plays a role in healthy status acquisition and maintenance [50,55]. As a consequence, a microbial dysbiosis may contribute to diseases development and may provide novel actionable targets, not only for disease monitoring, but especially for the development of novel therapies [50].

As the costs of NGS continue to decrease, it is conceivable to hypothesize that these (and other) applications will become even more common and will be part of clinical practice. It has to be noticed that clinical and research studies require different pipelines; indeed, clinical studies need a lot of validation, remain rigid and are concerned only with findings that are actionable, whereas research studies are more fluid and concerned with discovery. Moreover, NGS technologies have accustomed us to rapid developments. For instance, an old limitation of NGS was its limited reads length, currently exceeded by an increased reads length capability on one side, and the availability of ad hoc designed assembly tools, on the other. Novel DNA sequencing techniques promise to further improve this aspect [2,7]. For example, nanopore-based sequencing chemistries have the double advantages of being able to avoid the amplification of libraries (and the related errors) and to allow the sequencing of very long reads (up to 950 kb) [56]. Once the accuracy of these methods is increased and the error-rate minimized, we will observe a novel revolution in the DNA sequencing field and a further reduction of the sequencing costs/genome [2].

Besides the above-mentioned improvements, in a similar manner, high-throughput mass spectrometry (MS) platforms have also been developed to exploit in depth the whole proteome and/or metabolome of cells, biological fluids and tissues [2,57]. Representing the final products of cellular processes, proteins and metabolites studies, also in combination with other -omic approaches, have the potential to further clarify pathogenetic mechanisms and highlight additional biomarkers. Mass spectrometry improvements allow the simultaneous analysis of multiple peptides/metabolites and also untargeted approaches for novel molecules detection [2,57]. However, peptides/metabolites identification is based on the comparison of the results obtained in the analyzed sample with respect to specific databases that still present limitations, especially for metabolomic evaluations. As further technological progress will be achieved, both proteomic and metabolomics profiling may integrate genomic data for a better diagnostic and prognostic classification. For more details regarding proteomic and metabolomics technologies, the interested reader is referred to specific papers on these topics [2,57].

## 3. Big Data Production, Big Data Analysis and Data Integration Methods

The term ‘big data’ indicates a huge amount of structured or unstructured data not analyzable by using traditional technologies, and characterized by great variety, high production speed, and extreme variability [58,59]. Technological advances in -omic sciences have brought them into the big data domain [60].

In recent years, we have been overwhelmed by a real technological escalation, far from the expected logarithmic trend based on Moore’s laws [2,39]. Currently, molecular protocols for high-throughput analyses have been well established, extensively validated and simplified to analyze an increasing number of samples in even less time and costs [2,7,10]. Liquid handling platforms for libraries preparation have also been developed to further optimize the samples preparation step by reducing both inter-samples variability and the hands-on time. The direct consequence of this phenomenon is that the more we are able to sequence fast, high-quantity bases at a high level of accuracy and lower cost, the more we accumulate data. The last few years have shown that our ability to generate data supersedes our possibility to analyze and interpret them [2,5,6,7,8,9,10]. Consider that a sequencing run is able to generate hundreds of gigabytes at a time and it has been estimated that in the next ten years we may sequence up to two billion human genomes [61]. Considering the rapid development of novel technological platforms for data production, it is also possible that this number may be currently underestimated. A couple of years ago, an interesting report already compared genomics to other big data classical domains, and considering four typical features of a dataset’s life-cycle (i.e., acquisition, storage, distribution, and analysis), defined genomic as a “four-headed beast” since its needs overcome that of all the others [60].

This big data production has imposed the development and validation of specific bioinformatic tools for their analysis, starting from quality check, background noise minimization and reads normalization [62]. Data analysis and interpretation now represent the most important challenge to be addressed when approaching high-throughput analyses. Today, NGS methods offer a plethora of applications investigating different biological systems, both in a deeper focused and/or genome-wide manner [2,5,6,7,8,9,10,62]. Each of these procedures requires specific validated pipelines to address a specific biological question, i.e., identify disease-related mutations, obtain a differential expression analysis, or define the microbiome composition [2,5,6,7,8,9,10,62,63]. These operations not only need highly qualified, specific expertise to develop highly sensitive (and preferably easy-to-use) tools for data management and analysis, but also need cooperative efforts to establish quality guidelines ensuring data comparison among different datasets and different laboratories worldwide [9,64]. Indeed, if each laboratory uses its own pipeline to analyze its own results, the risk of finding everything and its exact opposite, without the possibility of comparing the results obtained by different studies, may become a reality. Reproducibility is a key feature of scientific research; high-throughput data are challenging in this regard due to the high variability of the samples analyzed and/or of the experimental procedures, and the complexity of the data and the use of not properly validated and/or standardized pipelines. Statistician-derived methods may be useful in this context by supporting experimental design and reproducibility, preprocessing, structure learning, and data integration [65]. The information is in the data: the methodology for their correct interpretation must be widely validated and standardized to ensure laboratory data harmonization and be sure that significant differences in a specific sample, or in a population, are really due to a relevant biological alteration and not to biases attributable to the used analytical approaches (both at molecular and bioinformatics levels). The re-analysis with updated pipelines of samples previously reported as “negative”, and the management of the so-called incidental findings are other relevant hot topics in this field [42,66,67]. Both of these aspects also require caution and shared guidelines.

In addition, the continued development of novel NGS-based strategies requires the continued availability of ad hoc pipelines able to overcome possible methodological limitations and highlight the biologically relevant information. For example, the recent introduction of methodologies able to achieve a single cell resolution gave an unprecedented opportunity to study cellular heterogeneity at different levels. This, in turn, requires computational methods able to overcome some criticisms, such as the systematic noise, the complexity of the data, and the need of validation techniques [68,69]. Despite several computational solutions, an important bottleneck is currently represented by the need to overcome biological and technical variability in single cell data [69]. Since a number of papers addressing these issues are being published, it is conceivable to suppose that current challenges and limitations in single cell analysis will soon be overcome, opening the way to new applications and opportunities [70,71,72].

Furthermore, specific comprehensive databases are required to compare the results obtained in the samples of interest and infer biologically and clinically relevant information [2,5,6,7,8,9,10,62,64]. The continued evolution of NGS applications also requires the corresponding and continued evolution of bioinformatic instruments (including the update of the reference databases). Limitations in the reference databases also negatively impact the interpretations of data derived from genome-wide proteomic or metabolomics analyses, whose potential seems now to be, consequently, underestimated [2,57]. In addition, the increasing throughput of the sequencers imposes the need for tools that are able to manage a huge amount of data. Consequently, an additional problem is the storage of the generated data. Despite the technological advances in big data production, their management (including data storage and the computational resources required for their analysis and interpretation) is still expensive [73]. A promising solution for -omics data handling is represented by cloud computing. These systems are based on virtual, web-based solutions, able to use multiple computational resources simultaneously, escalating the computational power in respect to local-based servers [73]. The large diffusion of cloud computing is partly due to the availability of infrastructures, such as Hadoop [74]. Hadoop is an open-source software that allows the processing of large datasets by distributing them across multiple computer nodes; thus, it is particularly suitable for bioinformatic purposes [75]. Depending on the services offered, in terms of the level of functionality given to the user by the cloud provider, cloud computing-based solutions for big data manipulation can be classified as Data as a Service (DaaS), Software as a Service (SaaS), Platform as a Service (PaaS), and Infrastructure as a Service (IaaS) [75]. In addition to integrating data and specific tools for their analysis, the bioinformatics cloud should provide technologies for high-speed data transfer, allow the development of customized pipelines by the users, and be publicly accessible. Further, once data have been properly stored, we need a system to manage them in order not only to efficiently archive data, but also to make them easily available to the user on demand. In particular, different kinds of NoSQL (Not only Structured Query Language) databases have rapidly emerged showing their benefits over traditional relational databases in the speed of data storage, indexing, and query retrieval [76]. Finally, LIMS (laboratory information management system) solutions support standardized data management and tracking systems [77,78,79]. Large repositories, such as TCGA [80] and CBioPortal [81], represent successful examples regarding big data analysis, management, integration, and sharing.

While a lot of companies are offering cloud-based solutions for data storage, ethical concerns are emerging regarding their safety and properties [82]. Similar issues are also emerging with regard to data sharing [2,10]. The sharing of research data offers the unique opportunity to increase knowledge by avoiding unnecessary duplications and obtaining novel, useful information from the re-analysis of the same datasets. However, it imposes several challenges of ethical, cultural, legal, financial, and technical nature [83]. Even if a couple of years ago the Regulatory and Ethics Working Group of the Global Alliance for Genomics and Health proposed a standardized model for data sharing [84], its application seems far away.

Considering the escalation of high-throughput technologies and their exponentially increasing ability to generate high-quality datasets of huge size, big data algorithms and high-performance computing (HPC) systems are needed for large-scale analyses [59,61,73,85]. Schmidt et al. recently reviewed big data analysis techniques for NGS data and HPC solutions and hypothesized a shift from model-driven to data-driven science [61]. This assumption requires caution since, if brought to its extreme consequences, the risk is to look just at data and not at biological systems.

Another important issue is related to the need of instruments for data integration. Indeed, big data production related to -omic sciences has led to the onset of the so-called ‘systems biology’. Systems biology proposes a holistic view based on the integration of multidisciplinary data to infer mechanistic associations and gain insights into biologic processes, including complex human diseases, in view of personalized medicine [73]. However, the high-throughput technologies described above are often focused on a specific -omic network (i.e., DNA, RNA, proteins, or metabolites). Consequently, even if these approaches are very effective, they miss a comprehensive view since they focus on a single system at a time. This limitation may contribute to the gap between the ability to produce huge amounts of data and the difficulty to correlate these data to complex phenotypes and to predict their outcomes. Since high-throughput analyses coupled with bioinformatics are becoming routine procedures, it is easy to hypothesize that the future direction will be the integration of multi-omics data. By integrating multiple sources of data, it is possible to highlight information that may be underestimated in the analysis of just one -omic level, and it is possible to reinforce the strength of some associations by confirming them at multiple levels [86]. The identification of specific interactions across -omic levels may shed light on the molecular mechanisms underlying human diseases and support the identification of biomarkers for disease risk prediction or monitoring. However, this process is currently still complex and difficult due to several factors, i.e., different sources of data and different data formats, the lack or redundancy of databases, and the lack of data standards [87]. Thus, different strategies are being proposed to efficiently integrate the information concerning the complex relationships across multi-omics levels by using systems genomic approaches [86,88,89,90,91,92,93]. Based on the algorithms used, data integration methods can be classified into three categories: unsupervised, supervised, and semi-supervised [93]. Briefly, unsupervised methods use different approaches to cluster objects into different categories based on their biological profiles; supervised methods start from known labels (i.e., disease or healthy) to predict the related patterns and assign the unlabeled data to each of them; and, finally, semi-supervised methods, often graph-based, use both labeled and unlabeled samples to develop learning algorithms based on similarity networks [93]. In this context, Yugi et al., proposed a “trans-omic” analysis to obtain a global network from multi-omics data and applied it to three case-studies showing the potentialities of this approach, even if technological improvements, including validation strategies, are required [90]. Dimension reduction approaches have also been used for the analysis of multiple data sets [92]. These methods calculate the most valuable linear relationships able to explain the correlation across data sets and can also evaluate the variability effects of outliers [92].

Machine learning and system genomics (MLSG)-based approaches are also showing their reliability in the identification of genotype–phenotype relationships, as a result of the integration of multiple data from multi-omics analyses by using predictive algorithms and data mining [94,95]. Indeed, machine learning employs predictive algorithms able to recognize specific patterns from complex data and to learn from them in order to use this knowledge to make reliable predictions. Thus, this kind of analysis requires as its first step the design of a model, starting from known datasets used as examples to generate the associations, before it can be used to make predictions [94,95]. In the field of MLSG, different software for the prediction of genotype–phenotype relationships from multi-omics data have been developed based on different methods, i.e., different models to predict significant associations, showing the potential of MLSG in predicting diseases outcome [94]. Despite this great potential as a key technology in supporting clinical decisions, machine learning is still not widely used [96]. This is partly due to the lack of the required skills and expertise of life sciences researchers that are often unable to infer biologically relevant information from the huge amount of data they produce. The need for multidisciplinary teams has already been postulated in recent years. Alyass et al., for example, pointed out that the road to personalized medicine requires a strong, or we can say a revolutionary, integration between traditionally well-separated disciplines [73]. This is also the reason why many efforts are being made to make computational instruments easy-to-use for inexperienced users. Luo et al. developed Automated Machine-Learning (Auto-ML) to automate the whole machine learning process and support patients’ outcome predictions [96]. In this context, graphical user interfaces (GUI) may support inexperienced users in respect to a command line interface (CLI) [97].

Based on all the above, it is easy to predict that the next few years will feature further development of statistical, mathematical and information technology (IT) instruments in the -omic context. This will completely change the care process and the concept of medicine, and will also require careful regulation to avoid the risks related to a data-centric view.

## 4. Conclusions

**Figure 1:**
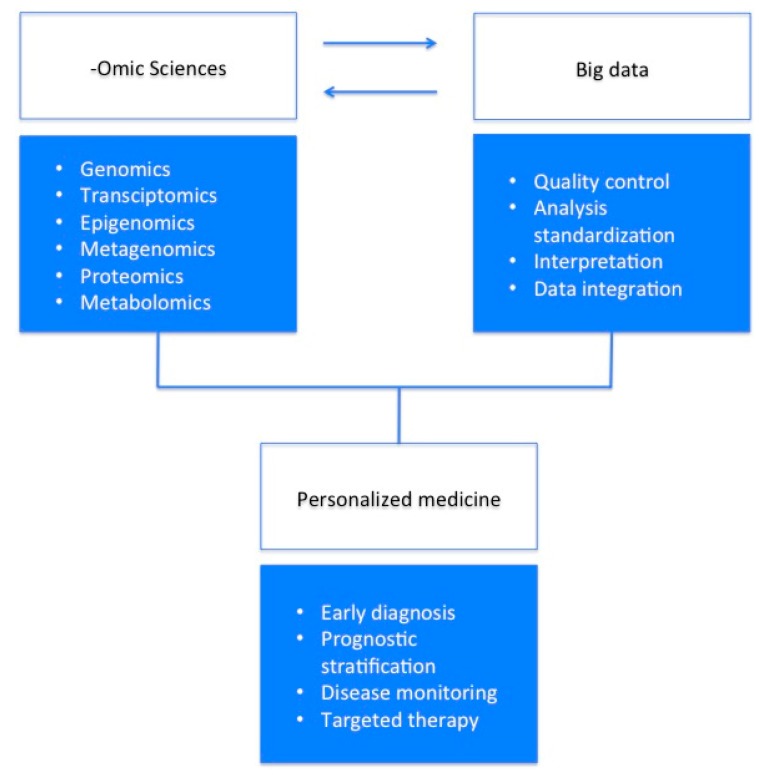
The integration between different -omic sciences and validated and standardized tools for big data analysis will bring personalized medicine into real clinical practice.

Since it has been estimated that the cost of the sequencing will continue to decrease, whole-genome sequencing may become a routine clinical practice to obtain clinically relevant information for the correct and early diagnosis, to determine the most proper therapy, and for disease monitoring. The integration of these data with those derived from other -omic approaches will shed light on the mechanisms underlying human diseases and will allow the identification of novel biomarkers for the diagnosis and monitoring of diseases, as well as actionable targets for specific therapies in view of an even more personalized medicine approach (Figure 1).

Personalized medicine means that medical decisions are customized to each individual based on specific biomarkers obtained from multi-omics data.

The availability of high-throughput methods coupled with tools for big data analysis and integration, and machine learning-based approaches has the potential to bring personalized medicine into real medical practice. Indeed, the access to an individual’s genomic content will provide information not only on the underlying disease but also may highlight actionable target for specific therapies, and infer prediction regarding specific outcomes. To bring this model into clinical practice, issues concerning big data production and interpretation need to be assessed. Once we are able to validate and establish shared pipelines for the accurate analysis of high-throughput-derived data, also including the ethical aspects to regulate privacy issues and data sharing, we may be able to fight the data struggle.
